# The Surface Proteome of Bovine Unsexed and Sexed Spermatozoa

**DOI:** 10.3390/ani15040484

**Published:** 2025-02-08

**Authors:** Patrícia Pinto-Pinho, Joana Quelhas, Francis Impens, Sara Dufour, Delphi Van Haver, Graça Lopes, António Rocha, Rosário Pinto-Leite, Margarida Fardilha, Bruno Colaço

**Affiliations:** 1 Centre for the Research and Technology of Agro-Environmental and Biological Sciences, University of Trás-os-Montes and Alto Douro, 5000-801 Vila Real, Portugal; 2 Laboratory of Signal Transduction, Department of Medical Sciences, Institute of Biomedicine, University of Aveiro, 3810-193 Aveiro, Portugal; juquelhas@gmail.com (J.Q.); mfardilha@ua.pt (M.F.); 3 Laboratory of Genetics and Andrology, Hospital Center of Trás-os-Montes and Alto Douro, E.P.E., 5000-508 Vila Real, Portugal; mlleite@chtmad.min-saude.pt; 4 Experimental Pathology and Therapeutics Group, IPO Porto Research Center, Portuguese Institute of Oncology of Porto Francisco Gentil, E.P.E., 4200-072 Porto, Portugal; 5 Department of Veterinary Clinics, Institute of Biomedical Sciences Abel Salazar, University of Porto, 4050-313 Porto, Portugal; mdlopes@icbas.up.pt; 6 Department of Veterinary Sciences, University School Vasco da Gama, 3020-210 Coimbra, Portugal; 7 VIB Proteomics Core, B-9052 Ghent, Belgium; francis.impens@vib-ugent.be (F.I.); sara.dufour@vib-ugent.be (S.D.); delphi.vanhaver@vib-ugent.be (D.V.H.); 8 VIB-UGent Center for Medical Biotechnology, Flemish Institute for Biotechnology, B-9052 Ghent, Belgium; 9 Department of Biomolecular Medicine, Ghent University, B-9052 Ghent, Belgium; 10 Department of Imuno-Physiology and Pharmacology, Institute of Biomedical Sciences Abel Salazar, University of Porto, 4050-313 Porto, Portugal; amrocha@icbas.up.pt; 11 Animal and Veterinary Research Centre, University of Trás-os-Montes and Alto Douro, 5001-801 Vila Real, Portugal; bcolaco@utad.pt

**Keywords:** proteomics, sperm surface proteome, sex-specific membrane proteins, sperm sexing

## Abstract

Current sperm sexing methods are costly and largely restricted to cattle, while immunological techniques targeting sex-specific membrane proteins may offer more economical alternatives. To advance these methods, understanding the proteomic differences between the cell membranes of X- and Y-chromosome-bearing spermatozoa is essential. This study aimed to characterize the cell surface proteome of bovine sperm and identify potential targets for sperm sexing through LC-MS/MS analysis. Cell surface protein lysates were extracted from unsexed, X-sperm (BX), and Y-sperm (BY) samples via biotinylation. Promising targets were identified through functional annotation (UniProt, eggNOG-mapper v.2.1.7) and topology prediction (DeepTMHMM v.1.0.13). Additionally, statistical overrepresentation (PANTHER 18.0) and orthology analyses were performed. Excluding contaminants, 130 proteins were detected, of which 64 proteins were detected in the BX samples and not in the BY samples. Of these, five transmembrane proteins stood out as potential X-sperm targets (ADAM2, ATP11C, DG1, MCT1, and PMCA4). They were identified as potential cell surface targets, based on GO terms and topology predictions, detected in at least two replicates of the BX samples, and shown to share orthology with other livestock species. These findings enhance our understanding of bovine sperm proteomics; however, further validation is required to confirm the utility of these five proteins in sperm sexing technologies.

## Simple Summary

Current sperm sexing methods are expensive and mainly used in cattle. Immunological techniques targeting sex-specific proteins could be a more cost-effective alternative. This study explored differences in surface proteins between X- and Y-chromosome-bearing bovine spermatozoa to identify potential targets for sperm sexing by LC-MS/MS analysis. Out of 130 identified proteins, 64 were unique to X-sperm, with 5 transmembrane proteins (ADAM2, ATP11C, DG1, MCT1, and PMCA4) showing promise as markers for X-sperm. While these findings advance knowledge of bovine sperm proteomics, further research is needed to validate these proteins for sperm sexing.

## 1. Introduction

The use of semen enriched with sex-selected spermatozoa stands out as a crucial strategy for producers seeking precise control over breeding outcomes [1]. However, the widespread availability of sexed semen for rabbits, pigs, goats, rams, and horses would provide significant advantages in terms of profitability and overall animal welfare across diverse livestock industries [2,3].

Sperm sexing techniques like flow cytometry and gender ablation are primarily employed in cattle due to high costs that make them mostly infeasible for implementation in other species, besides adversely affecting semen quality [4,5,6,7]. Immunological sperm sexing methods present a promising alternative. Although most X and Y gene expression is suppressed during meiotic sex chromosome inactivation, many X and Y genes are reactivated or specifically activated in haploid germ cells after meiosis (reviewed in [8]). Even though X- and Y-bearing sperm cells develop simultaneously due to their interconnections during spermatogenesis—maintaining similarities in origin, maturation, and functions—it is described that a complete sharing of gene products between them does not occur (reviewed in [9]). Moreover, while spermatozoa were long thought to be incapable of transcription and translation, various types of coding and non-coding RNAs have been identified in human sperm (reviewed in [10]). There is also evidence that ejaculated sperm are capable of protein translation during the final maturation steps before fertilization, in a process dependent on capacitation (reviewed in [11]). These observations open the door to the existence of proteins specific to X- and Y-chromosome-bearing spermatozoa (X-/Y-sperm), as already described [12,13,14]. Therefore, by targeting sex-specific membrane proteins accessible from the cell surface, immunological sperm sexing methods may offer a less invasive and cheaper alternative [7]. Nevertheless, a deeper understanding of the proteomic differences between X- and Y-sperm is required for the improvement or development of such techniques.

Given the commercial availability of bovine sexed semen, some proteomic studies have been undertaken in the past few years to explore the potential differential proteomic profiles of bovine semen enriched in X-sperm (X-semen, BX) and Y-sperm (Y-semen, BY) [12,15,16,17,18,19]. Only two of these studies specifically concentrated on the sperm membrane proteome of *Bos taurus* and *Bos indicus* bulls [12,18].

Aiming to expand the current understanding of sperm membrane proteomics in both unsexed (BU) and sexed spermatozoa and identify a catalog of potential cell surface target proteins for sperm sexing, a label-free liquid chromatography–tandem mass spectrometry (LC-MS/MS) analysis was conducted on the cell surface proteome of BU, BX, and BY samples after enriching cell surface proteins through a biotinylation approach. Cross-referencing these findings with sperm proteomic studies conducted in various species has the potential to reveal common protein targets specific to X- or Y-sperm.

## 2. Materials and Methods

### 2.1. Bovine Semen Samples Acquisition

For the BU condition and negative control (BC), fresh unsexed semen of a 2-year-old Limousin bull was used, provided by Lusogenes (Aveiro, Portugal). For the BX condition, a total of 110 cryopreserved straws of 2 million X-sorted sperm (Holsteins-Frisian; purity > 90%) were purchased from Genex Cooperative (Shawano, WI, USA) and Genoglobal (Vila do Conde, Portugal), and pooled. For the BY condition, a total of 110 cryopreserved straws of 2 million Y-sorted sperm (Angus and Asturian; purity > 90%) were purchased from Genex Cooperative (Shawano, WI, USA) and Genoglobal (Vila do Conde, Portugal), and pooled. All the experimental procedures were performed under DR 1.ª série- N.º 1/2019 of January 2019, the European Union Directive 2010/63/EU for animal experiments, and the companies’ guidelines.

### 2.2. Semen Quality Assessment

The fresh semen sample used for BX and BU was analyzed after collection at Lusogenes‘ laboratory by a trained technician, assessing the density, mass motility, individual motility, volume, concentration (Neubauer improved chamber, 0.100 mm, 0.0025 mm^2^, Hirschmann EM Techcolor), and color of the semen sample. The sample exhibited high density, with a mass motility score of 4 (on a scale from 1 to 4, as per [20]), individual motility of 80%, a volume of 2.5 mL, a concentration of 199.5 × 10^6^ spermatozoa/mL, and a milky-white color. The sperm sample was used, approximately, 90 min after collection for BU and BC, and for BX and BY immediately after thawing in a water bath at 37 °C for 30 s. For the frozen–thawed sexed semen samples (BX and BY), only individual motility and concentration were assessed. Both pooled samples met the quality standards set by the commercial suppliers. Moreover, it was assumed that all samples were representative of the population of bulls suitable for breeding in semen collection stations.

### 2.3. Biotinylation and Isolation of Cell Surface Proteins

To identify surface proteins of BX, BY, BU, and BC samples, about 72 × 10^6^ spermatozoa were used per technical replicate (n = 3 per condition). After one wash with room temperature PBS 1X to remove the sperm medium (450× *g*, 3 min), the cell surface proteins were biotinylated and isolated with the Pierce^TM^ Cell Surface Protein Biotinylation and Isolation Kit (A44390, Thermo Scientific^TM^, Waltham, MA, USA), according to slightly modified manufacturer’s instructions. The spermatozoa pellets were washed twice with 2 mL of PBS 1X to ensure the removal of the seminal plasma. After biotinylation of the cell surface membrane proteins with EZ-LinkTM Sulfo-NHS-SS-Biotin, the samples were washed twice again with 2 mL of TBS 1X (450× *g*, 3 min). Then, the spermatozoa were lysed with the lysis buffer supplemented with protease inhibitors (HaltTM Protease Inhibitor Cocktail, 1861278, Thermo Scientific^TM^, Waltham, MA, USA) during a 60 min incubation on ice. Labeled proteins were then immobilized in NeutrAvidin^TM^ Agarose and washed five times with wash buffer, followed by three more washes with 50 mM HEPES (pH 8.0). The labeled proteins were finally eluted with 100 µL of 8 M urea in 50 mM HEPES (pH 8.0) and 100 µL of 20 mM DTT, after an incubation of 45 min, at room temperature, with end-over-end mixing on a rotator. The flowthrough was stored at −80 °C prior to further proteomics sample preparation. For the negative control (BC, n = 3), the same protocol was followed, but cells were incubated with PBS instead of the biotin solution.

### 2.4. LC-MS/MS

The shotgun proteomic analysis was performed at the Flemish Institute for Biotechnology (VIB, Ghent) under the scope of an EPIC-XS project (project EPIC-XS-0000186).

#### 2.4.1. Sample Preparation

The samples were homogenized by sonication using a Diagenode Bioruptor Plus instrument with the following settings: high-intensity power output, 10 cycles of 30 s ON/30 s OFF pulses, and a +10 °C water bath. Proteins were reduced by the addition of 15 mM DTT and incubation for 30 min at 55 °C and then alkylated by the addition of 30 mM iodoacetamide and incubation for 15 min at room temperature in the dark. Samples were further diluted with 20 mM HEPES pH 8.0 to a final urea concentration of 4 M and proteins were digested with 0.5 µg LysC (Wako) (1:100, *w*/*w*) for 4 h at 37 °C. Samples were again diluted to 2 M urea and digested with 0.5 µg trypsin (Promega, Fitchburg, WI, USA) (1:100, *w*/*w*) overnight at 37 °C. The resulting peptide mixture was acidified by the addition of 1% trifluoroacetic acid (TFA) and cleaned up with Phoenix clean-up cartridges (PreOmics, Planegg, Germany) according to the manufacturer’s protocol and purified peptides were dried completely in a rotary evaporator. Dried peptides were dissolved in 100 µL 50 mM triethylammonium bicarbonate (TEAB) and 130 IUB mU Peptide-N-Glycosidase F (PNGase F) was added for deglycosylation at 37 °C overnight. Peptides were acidified with 1% TFA to lower the pH below 3 and desalted on reversed-phase (RP) C18 OMIX tips (Agilent, Santa Clara, CA, USA). The tips were first washed 3 times with 100 µL pre-wash buffer (0.1% TFA in water/acetonitrile (ACN, 20:80, *v*/*v*)) and pre-equilibrated 5 times with 100 µL of wash buffer (0.1% TFA in water) before the sample was loaded on the tip. After peptide binding, the tip was washed 3 times with 100 µL of wash buffer and peptides were eluted twice with 100 µL elution buffer (0.1% TFA in water/ACN (40:60, *v*/*v*)). The combined elutions were transferred to HPLC inserts and dried in a vacuum concentrator.

#### 2.4.2. LC-MS/MS and Data Analysis

Peptides were re-dissolved in 20 µL loading solvent A (0.1% trifluoroacetic acid in water/acetonitrile (ACN) (98:2, *v*/*v*)) of which 8 µL was injected for LC-MS/MS analysis on an Ultimate 3000 RSLCnano system in-line connected to a Q Exactive HF mass spectrometer (Thermo Scientific^TM^, Waltham, MA, USA). Trapping was performed at 20 μL/min for 2 min in loading solvent A on a 5 mm trapping column (Thermo scientific, 300 μm internal diameter (I.D.), 5 μm beads). The peptides were separated on a 250 mm Waters nanoEase M/Z HSS T3 Column, 100 Å, 1.8 µm, 75 µm inner diameter (Waters Corporation, Milford, MA, USA) kept at a constant temperature of 45 °C. Peptides were eluted by a non-linear gradient starting at 1% MS solvent B, reaching 33% MS solvent B (0.1% FA in water/acetonitrile (2:8, *v*/*v*)) in 100 min, 55% MS solvent B (0.1% FA in water/acetonitrile (2:8, *v*/*v*)) in 135 min, 70% MS solvent B in 145 min followed by a 10 min wash at 70% MS solvent B and re-equilibration with MS solvent A (0.1% FA in water). The mass spectrometer was operated in data-dependent mode, automatically switching between MS and MS/MS acquisition for the 12 most abundant ion peaks per MS spectrum. Full-scan MS spectra (375–1500 m/z) were acquired at a resolution of 60,000 in the Orbitrap analyzer after accumulation to a target value of 3,000,000. The 12 most intense ions above a threshold value of 15,000 were isolated with a width of 1.5 m/z for fragmentation at a normalized collision energy of 30% after filling the trap at a target value of 100,000 for a maximum of 80 ms. MS/MS spectra (200–2000 m/z) were acquired at a resolution of 15,000 in the Orbitrap analyzer.

Analysis of the mass spectrometry data was performed using MaxQuant (v. 2.0.3.0) with mainly default search settings including a false discovery rate set at 1% on peptide and protein level. Spectra were searched against the bovine reference proteome used (version 11 2021, UP000009136 [21]). The mass tolerance for precursor and fragment ions was set to 4.5 and 20 ppm, respectively, during the main search. Enzyme specificity was set as C-terminal to arginine and lysine, also allowing cleavage at proline bonds with a maximum of two missed cleavages. Variable modifications were set to oxidation of methionine residues, acetylation of protein N-termini, and deamidation of asparagine and glutamine residues. Matching between runs was enabled with a matching time window of 0.7 min and an alignment time window of 20 min. Only proteins with at least one unique or razor peptide were retained (Supplementary Spreadsheet S1.1). Proteins were quantified by the MaxLFQ algorithm integrated in the MaxQuant software (v. 2.0.3.0). A minimum ratio count of two unique or razor peptides was required for quantification (Supplementary Spreadsheet S1.2). Further data analysis of the shotgun results was performed with an in-house R script, using the protein groups output table from MaxQuant. Reverse database hits were removed, LFQ intensities were log2 transformed, and replicate samples were grouped. Proteins with fewer than two valid values in at least one group were removed and missing values were imputed from a normal distribution centered around the detection limit (package DEP) [22], leading to a list of 149 quantified proteins in the experiment. To compare protein abundance between pairs of sample groups (BU vs. BX, BU vs. BY, and BX vs. BY sample groups), statistical testing for differences between two group means was performed, using the package limma [23]. Statistical significance for differential regulation was set to a false discovery rate (FDR) of <0.05 and |log2FC| = 2 (Supplementary Spreadsheet S1.3). These statistical tests revealed 15 proteins downregulated in the BX samples compared to the BU samples, 37 downregulated in the BY samples compared to the BU samples, and 1 protein upregulated in the BX samples compared to the BY samples. After removal of 19 contaminants, these significantly regulated proteins together with proteins that were completely absent in either the BX or BY samples were plotted in a heatmap after non-supervised hierarchical clustering of z-scored protein log_2_ LFQ intensities (Supplementary Spreadsheet S1.4).

The mass spectrometry proteomics data have been deposited to the ProteomeXchange Consortium via the PRIDE [24] partner repository with the dataset identifier PXD048616.

### 2.5. Characterization of the Proteome Profile of Bovine (Un)sexed Spermatozoa

For subsequent analyses, the ‘majority of protein identifiers’ (IDs) of each protein group were used to avoid accidental hits to a protein group and, from these, only one protein ID per group was considered (designated as ‘Unique protein ID’). The selected protein ID always corresponded to the first reviewed entry of each protein group or, if none of the entries of the protein group were reviewed, the first one was selected.

#### 2.5.1. Functional Annotation

The protein sequences were functionally annotated by combining information retrieved from the UniProt database ([25], accessed on 9 June 2022) and one-to-one fast orthology assignments using the eggNOG-mapper v.2.1.7 tool ([26], accessed on 9 June 2022), as described in [27]. Briefly, the gene name, protein name, length, Gene Ontology (GO) IDs, and chromosome associated with each protein entry were obtained from UniProt using the retrieve/ID mapping tool. Additionally, gene names, descriptions, and experimentally validated GO IDs were obtained from eggNOG, with consideration given to a taxonomic scope auto-adjusted per query, a minimum hit bit-score of 60, and thresholds of 80% for identity, minimum query coverage, and minimum subject coverage.

**Figure 1:**
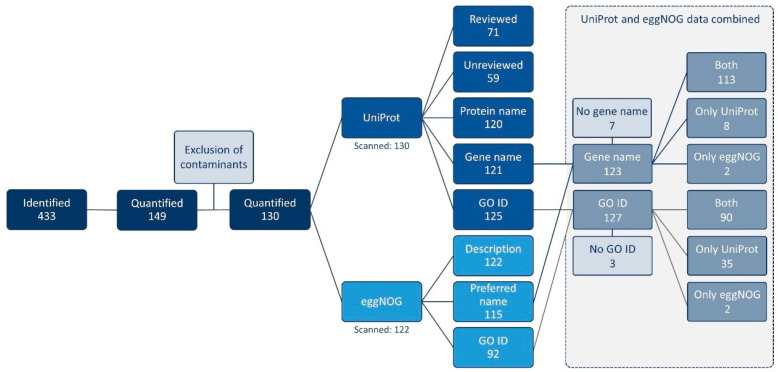
The schematization of the 130 detected proteins’ functional annotation by UniProt and eggNOG-mapper v2.1.7, concerning entry status, protein name, gene name, and Gene Ontology identifiers (GO ID).

Out of the 130 detected proteins, 71 (54.6%) had information manually verified by UniProt curators (Supplementary Spreadsheet S1.5). Utilizing the eggNOG-mapper v2.1.7 tool, a total of 122 entries were scanned (Supplementary Spreadsheet S1.6). By combining data from both tools, a total of 123 proteins were characterized with a gene name, and 127 had GO information. However, 2 proteins still lacked information on a description, protein, gene, and preferred names. Figure 1 provides a summary of the functional annotation results.

#### 2.5.2. Protein Topology Prediction

The topology of transmembrane proteins and the number of transmembrane regions (TMR) were predicted using DeepTMHMM v.1.0.13 ([28], accessed on 29 September 2022), as described in [27,29]. Proteins were classified as alpha-helical transmembrane (TM), beta-barrel transmembrane (BETA), or globular (GLOB). The presence of a signal peptide (SP) was also predicted, as well as which parts of the proteins’ sequence were outside or inside the cell membrane (Supplementary Spreadsheet S1.7).

#### 2.5.3. Statistical Overrepresentation Test

To assess whether the total number of quantified proteins, BX proteins, and BY proteins exhibited underrepresented or overrepresented biological process terms, molecular function terms, cellular component terms, protein classes, or pathways in comparison to the Reference Proteome Genome list of *Bos taurus*, a statistical overrepresentation test was conducted using PANTHER 18.0 (GO-Slim) [30]. The Fisher’s exact test with Bonferroni correction for multiple testing was employed and only the significantly under- and overrepresented terms were considered for analysis (*p*-value ≤ 0.05).

### 2.6. Identification of Possible Membrane Protein Targets for Sperm Sexing

A good target protein for an immunological sperm sexing method would be specific to X- or Y-sperm and accessible from the cell surface. Preferably, it should be a transmembrane protein. Integral membrane proteins are stable, embedded within the phospholipid bilayer, and may have one or more transmembrane domains, allowing accessibility from the cell surface [31]. In contrast, peripheral membrane proteins are associated with the lipid membrane either directly or indirectly via integral membrane proteins, and their association can be reversible in some cases [31].

Although a kit designed for the extraction of cell surface proteins was used, to improve the likelihood of identifying a potential target, proteins were further analyzed for their potential cellular localization and topology. This involved examining GO IDs retrieved from UniProt and associated with eggNOG, along with predictions of the protein’s topology using DeepTMHMM (Supplementary Spreadsheet S1.8).

Firstly, a total of fourteen GO IDs of interest, related to the plasma membrane, cell surface, and extracellular space were retrieved from the AmiGO 2 GENEONTOLOGY web application [GO:0005886 (plasma membrane, PM), GO:0005904 (PM), GO:0009986 (cell surface, CS), GO:0009928 (CS), GO:0009929 (CS), GO:0009897 (external side of plasma membrane, ESPM), GO:0031232 (extrinsic component of external side of plasma membrane, ECE-SPM), GO:0046658 (anchored component of plasma membrane, ACPM), GO:0031362 (anchored component of external side of plasma membrane, ACESPM), GO:0071575 (integral component of external side of plasma membrane, ICESPM), GO:0005887 (integral component of plasma membrane, ICPM), GO:0010339 (external side of cell wall, ESCW), GO:0031240 (external side of cell outer membrane, ESCOM), and GO:0005615 (extracellular space, ES)] ([32], accessed on 28 July 2022).

Then, an automated script (available at [33]) was used to generate a list with the union of both UniProt and eggNOG GO IDs associated with each protein and to indicate which proteins contained at least one of the fourteen GO IDs of interest. All proteins related to the plasma membrane were classified as “of interest”, meaning proteins with the GO term PM, CS, ESPM, ECESPM, ACPM, ACESPM, ICESPM, ICPM, ESCW, or ESCOM, as well as transmembrane proteins with the GO term ES. Of these, some were highlighted as potential targets accessible from the cell surface if associated with the plasma membrane and located on the external side of the membrane or predicted to be transmembrane, and if they were predicted to be transmembrane proteins associated with the extracellular space.

Therefore, after crossing the list of proteins of interest with the results of DeepTMHMM, a list of possible targets was created by considering all proteins with GO IDs for CS, ESPM, ECESPM, ACESPM, ICESPM, ESCW, and/or ESCOM, all proteins with GO IDs for PM, ACPM, and/or ICPM that were predicted to be transmembrane or had at least part of their sequence predicted to be outside the cell membrane, and proteins with the GO ID for ES that were predicted to be transmembrane proteins.

Lastly, we further highlighted potential targets specific to the BX or BY samples, i.e., detected in the BX samples but not in the BY samples, or vice versa. Special attention was given to those identified in at least 2 replicates of the BX or BY samples and predicted to be transmembrane proteins.

#### Orthology Analysis

For each identified target, we investigated the presence of orthologs in *Homo sapiens* (human), *Bos taurus* (bovine), *Ovis aries* (sheep), *Sus scrofa* (pig), and *Equus caballus* (horse), utilizing the list of orthologs from the NCBI database, Ensembl BioMart tool, and/or verifying local synteny [34]. The percentage of protein sequence similarity between bovines and each of these species was determined using the Align tool of the UniProt database [35].

## 3. Results

### 3.1. Mapping the Surface Proteome of Bovine (Un)sexed Spermatozoa

To investigate the surface proteome of bovine spermatozoa and identify possible targets of X-sperm and Y-sperm, we utilized a mass spectrometry-based proteome analysis coupled with biotinylation for the enrichment of cell surface proteins. Spermatozoa were treated with EZ-Link^TM^ Sulfo-NHS-SS-Biotin, lysed, and biotinylated proteins were immobilized in NeutrAvidin^TM^ Agarose columns. The isolated proteins were then digested with trypsin, and the resulting peptides were analyzed via label-free liquid chromatography–tandem mass spectrometry (LC-MS/MS). To validate the specificity of this approach, we quantitatively compared the proteome of three biological replicate samples of bovine unsexed spermatozoa processed with and without biotinylation.

**Figure 2:**
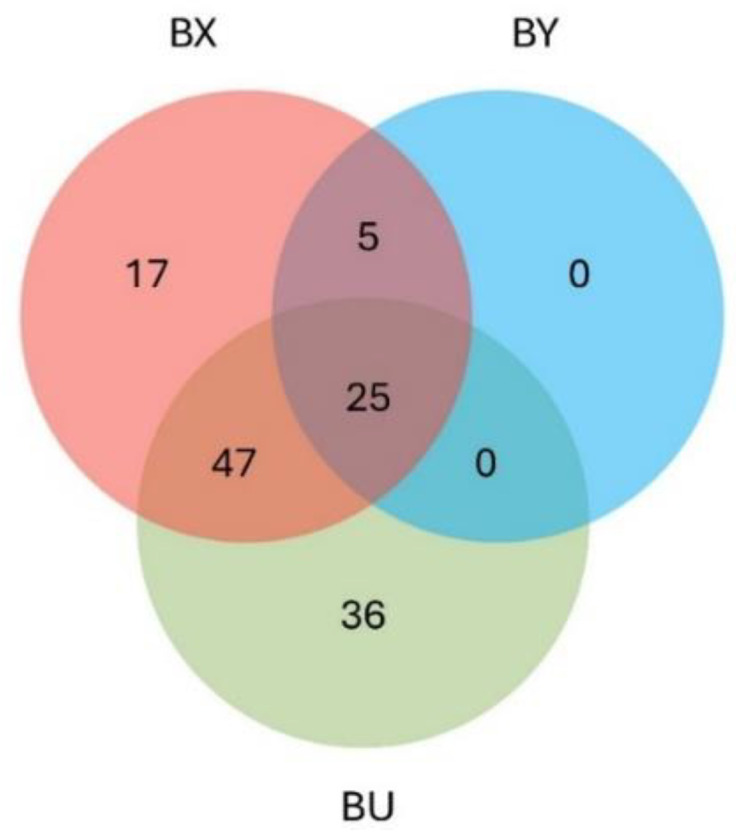
Venn diagram illustrating the number of detected proteins in at least one replicate of the unsexed semen (BU), X-semen (BX), and Y-semen (BY) conditions. No proteins were identified in the negative control (BC).

A total of 433 bovine proteins were identified (Supplementary Spreadsheet S1.1), of which 149 were reliably quantified (at least two or three valid values in one of the groups, listed in the Supplementary Spreadsheet S1.2). After excluding 19 contaminants, we further characterized the remaining 130 proteins by identifying their protein and gene names, topology, associated chromosomes, and GO terms (Supplementary Spreadsheets S1.5–S1.8). Of these 130 proteins, 108, 94, and 30 proteins were identified in at least one replicate of the BU, BX, and BY samples, respectively (Figure 2). No proteins were detected in the BC replicates, supporting their identification as bona fide cell surface proteins.

### 3.2. Functional Annotation of the Surface Proteome

**Figure 3:**
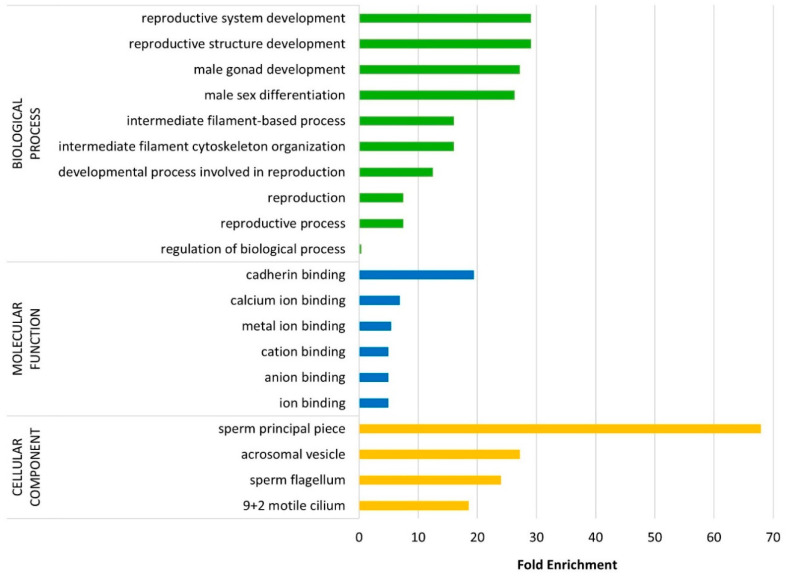
Summary of the up to 10 most overrepresented biological processes, molecular functions, and cellular components among the 130 quantified proteins, according to the statistical overrepresentation test performed with PANTHER 18.0 (GO-Slim, 117 entries mapped out of 130; *p*-value ≤ 0.05, Fisher’s exact test, Bonferroni correction for multiple testing).

To enhance our comprehension of potential enrichments in biological process, molecular function, and cellular component terms within the 130 detected proteins in comparison to the Reference Proteome Genome of Bos taurus, we performed an overrepresentation analysis using PANTHER 18.0. This analysis revealed ‘reproductive system development’ (FE = 29.11), ‘reproductive structure development’ (FE = 29.11), and ‘male gonad development’ (FE = 27.17) as the most enriched terms linked to biological process. Among the molecular function terms, ‘cadherin binding’ (FE = 19.40), ‘calcium ion binding’ (FE = 6.91), and ‘metal ion binding’ (FE = 5.45) were the most enriched, while ‘sperm principal piece’ (FE = 67.91), ‘acrosomal vesicle’ (FE = 27.17), and ‘sperm flagellum’ (FE = 23.97) were the three most overrepresented cellular components. The 10 most significant over- or underrepresented biological process, molecular function, and cellular component terms are presented in Figure 3.

The topology of the proteins was also predicted using the DeepTMHMM v.1.0.13 software. Out of the 130 detected proteins, 62.3% (n = 81) were predicted to be globular proteins and 17.7% (n = 23) were predicted as alpha-helical transmembrane proteins with the number of transmembrane regions ranging from 1 to 12. Among the transmembrane proteins, 6.9% (n = 9) were also predicted to have a signal peptide. Moreover, 20% (n = 26) were solely predicted to have a signal peptide (Supplementary Spreadsheet S1.7). Additionally, 40% of the proteins (n = 52) had GO terms associated with the plasma membrane and/or cell surface and cross-referencing with the topology prediction indicates that 30% (n = 39) are potentially accessible from the cell surface (Supplementary Spreadsheet S1.8).

**Figure 4:**
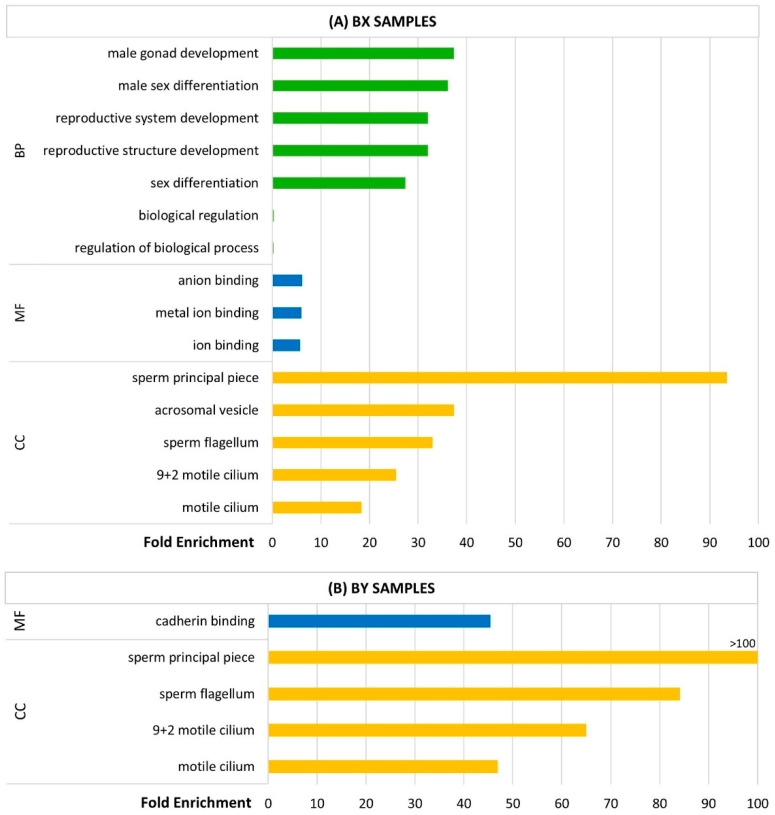
Summary of the up to 10 most overrepresented biological processes (BPs), molecular functions (MFs), and cellular components (CCs) among the (**A**) 94 quantified proteins identified in BX samples and (**B**) 30 quantified proteins identified in BY samples. The statistical overrepresentation test was performed with PANTHER 18.0 (GO-Slim, (**A**) 85 entries mapped out of 94; (**B**) 25 entries mapped out of 30; *p*-value ≤ 0.05, Fisher’s exact test, Bonferroni correction for multiple testing).

A statistical overrepresentation test was also conducted to analyze the 94 proteins detected in at least one replica of the BX samples (Figure 4A) and the 30 proteins detected in the BY samples (Figure 4B), using PANTHER 18.0.

The three most overrepresented biological process terms among the proteins detected in the BX samples were similar to the ones obtained for the total set of 130 detected proteins, encompassing ‘male gonad development’ (FE = 37.39), ‘male sex differentiation’ (FE = 36.19), and, with the same FE, both ‘reproductive structure development’ and ‘reproductive system development’ (FE = 32.05). Additionally, ‘anion binding’ (FE = 6.13), ‘metal ion binding’ (FE = 6.00), and ‘ion binding’ (FE = 5.71) were the most overrepresented molecular function terms. Similarly to the results obtained for the total set of proteins, the most overrepresented cellular component terms were the ‘sperm principal piece’ (FE = 93.48), ‘acrosomal vesicle’ (FE = 37.39), and ‘sperm flagellum’ (FE = 32.99).

On the other hand, when considering the proteins detected in the BY samples, no significantly enriched biological process terms were identified, and ‘cadherin binding’ was the only molecular function term found to be significantly enriched (FE = 45.41). Nevertheless, cellular component terms related to spermatozoa were once again found to be enriched, namely ‘sperm principal piece’ (FE > 100), ‘sperm flagellum’ (FE = 84.13), and ‘9 + 2 motile cilium’ (FE = 65.01).

### 3.3. Selecting Potential Membrane Protein Targets for Sperm Sexing

**Figure 5:**
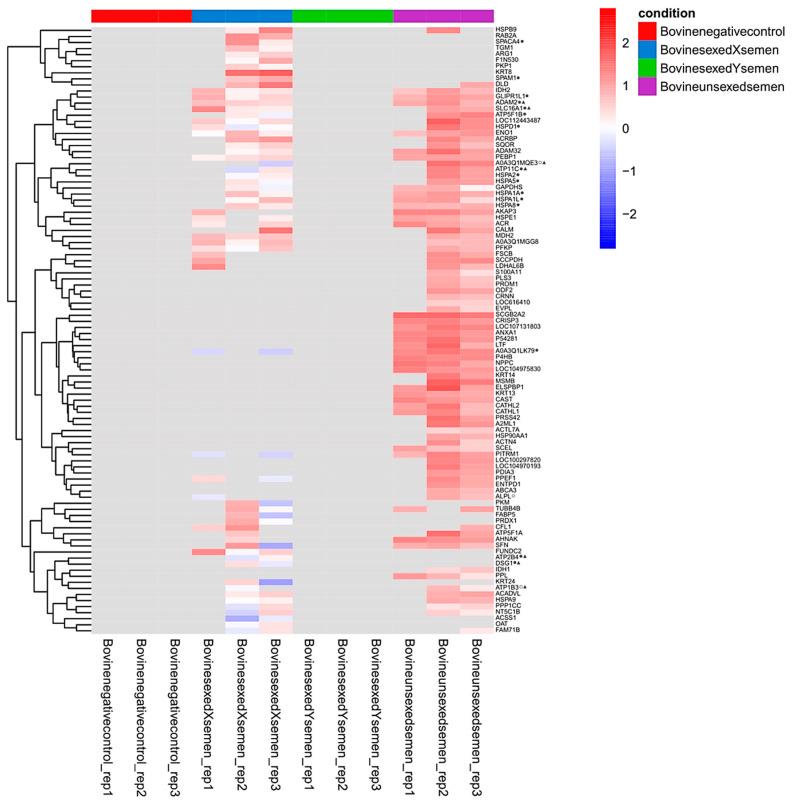
Heatmap visualizing 100 proteins that are either differentially regulated after non-supervised hierarchical clustering between conditions or completely absent in either the bovine X-semen (BX, blue) or bovine Y-semen (BY, green) samples. *Notably, none of the 100 proteins were identified exclusively in BY samples. Among these, a total of 15 proteins were significantly upregulated in BU samples compared to BX samples (SCGB2A2, CRISP3, LOC107131803, ANXA1, P54281, LTF, A0A3Q1LK79, P4HB, NPPC, LOC104975830, KRT14, MSMB, ELSPBP1, KRT13, and CAST), 35 proteins were upregulated in BU samples compared to BY samples (IDH2, GLIPR1L1, ADAM2, SLC16A1, ATP5F1B, LOC112443487, HSPD1, ENO1, ACRBP, ADAM32, PEBP1, HSPA1A, HSPA1L, AKAP3, ACR, SCGB2A2, CRISP3, LOC107131803, ANXA1, P54281, LTF, A0A3Q1LK79, P4HB, NPPC, LOC104975830, MSMB, ELSPBP1, KRT13, CAST, SCEL, PITRM1, LOC104970193, ENTPD1, AHNAK, and SFN), and 1 protein was upregulated in BX samples compared to BY samples (ADAM32). Among the 64 proteins identified in BX samples but not in BY samples, 19 were predicted to be accessible from the cell surface based on GO data and topology prediction. These proteins are marked with a filled circle (if identified in 2–3 BX replicates) or an empty circle (if identified in only 1 BX replicate). Of these, seven were predicted to be transmembrane proteins, making them structurally stable and more suitable for immunological sperm sexing techniques (highlighted with a triangle). Overall, five proteins stood out as potential targets, as they were identified in at least two BX replicates, absent in BY samples, and predicted to be transmembrane and surface-accessible (highlighted with both a filled circle and a triangle). Missing values are depicted in gray*.

To explore potential targets specific to X-sperm or Y-sperm, we focused on proteins that were differentially abundant between the BU, BX, and BY conditions after statistical testing (see Supplementary Spreadsheet S1.4 for a description of the proteins that differed significantly between conditions). In addition, we added proteins that were completely absent from all replicates of either the BX or BY condition. The intensities of a total of 100 proteins were plotted in a heatmap after non-supervised hierarchical clustering (Figure 5). The function of these proteins, as available in UniProt (accessed on 28 December 2024), is detailed in the Supplementary Spreadsheet S1.4.

The heatmap revealed three distinct groups of proteins: one group of proteins identified exclusively in the BX samples (n = 17), a second group of proteins identified in both the BX and BU samples (n = 47), and a third group of proteins identified exclusively in the BU samples (n = 36). No proteins specific to the BY samples were detected (Figure 5) and only one differed significantly between the BX and BY conditions, the disintegrin and metalloproteinase domain-containing protein 2 (ADAM2). Therefore, the first two groups of proteins (n = 64) were further studied to identify potential protein targets of X-sperm, accessible from the sperm surface, as these proteins were detected in the BX samples but not in the BY samples.

To achieve this, we cross-referenced our LC-MS/MS results with existing data on the cellular localization of these proteins, obtained through both databases and prediction software. We conducted a search for GO terms associated with each protein, using the UniProt database and fast orthology assignment using eggnog-mapper v.2.1.7. Additionally, we predicted their topology using DeepTMHMM v.1.0.13.

Of the 64 proteins analyzed, 26 (40.6%) had GO terms linking them to the plasma membrane and/or cell surface, 45 were predicted to be globular, 10 were predicted to be transmembrane proteins, of which 4 also had a signal peptide, and 9 were solely predicted to have a signal peptide (Supplementary Spreadsheet S1.4).

By integrating data on their cellular localization with topology predictions, 19 proteins were revealed to be potentially accessible from the cell surface (Table 1; for more details on their GO, protein topology, and function, please see the Supplementary Spreadsheet S1.4—proteins highlighted in green). Among these, five proteins stood out for a potential application in sperm sexing techniques, since they were predicted to be transmembrane proteins with at least one transmembrane region and were consistently identified in two or three replicates of the BX samples—ADAM2, phospholipid-transporting ATPase (ATP11C), desmoglein-1 (DG1), monocarboxylate transporter 1 (MCT1), and plasma membrane calcium-transporting ATPase 4 (PMCA4). It is worth noticing that ADAM2 was shown to be significantly upregulated in the BX samples compared to the BY samples, and that ATP11C is encoded by a gene located on the X chromosome.

A list of the five most promising proteins, along with the percentage of similarity between their protein sequences and the orthologous sequences in humans, rabbits, pigs, sheep, and horses, is provided in Table 2.

Among these five proteins, ADAM2 displays the lowest sequence similarities between bovine and other species. Despite the NCBI’s list of orthologs not identifying orthology for *ADAM2* across these species, each analyzed species has an ortholog for *ADAM2* according to the Ensembl database. However, the confidence level of orthology is not high, neither between bovine and rabbit nor between bovine and horse. Yet, if aligning the parts of the sequence of ADAM2 that are predicted to be outside the cell in bovines and rabbits, for example, it is possible to identify sets of 10–15 residues sharing 100% similarity (Supplementary File S1). Additionally, Ensembl has classified the orthology of *ATP11C* between bovines and rabbits as low confidence. Nevertheless, the NCBI also indicates orthology between these species, with *ATP11C* being preceded by *MCF2* in both. Moreover, aligning the five segments of the sequences of bovine ATP11C and rabbit ATP11C that are predicted to be outside the cell reveals a shared similarity ranging from 85.7% to 100% (Supplementary File S1).

## 4. Discussion

In this study, a shotgun proteomic analysis was conducted on cell surface proteins from bovine unsexed and sexed spermatozoa, extracted using a kit that has already proven effective for extracting cell surface proteins from spermatozoa [36,37].

For this experiment, due to technical constraints, only samples from beef bulls were used for the unsexed and BY conditions, while semen from dairy bulls was used for the BX condition [38,39]. The ideal scenario would involve using semen from both beef and dairy breeds, sexing it, and analyzing both the unsexed fraction and the sexed fractions for each, since the sperm proteome profile may vary between bulls of different breeds/genotypes, ages, or fertility level [40,41,42,43]. Specifically, it is described in the literature that diverse expression patterns for the same proteins or exclusive expression of some proteins have been described in different cattle breeds [44,45].

Moreover, both the BX and BY samples were cryopreserved, unlike the BU samples. Cryopreservation induces structural and biochemical damage to sperm, including oxidative and osmotic stress, altered lipid and protein configurations, decreased motility and viability, mitochondrial injury, and increased DNA fragmentation (reviewed in [46]). Namely, certain proteins may be differentially impacted by cryopreservation, resulting in changes in their abundance that could ultimately affect their detection [47]. Since the proteome of the fresh BX and BY samples might differ from that of their cryopreserved counterparts, it is important to account for these differences when interpreting results and selecting a target protein. This is particularly significant given that sex sorting techniques are typically applied to fresh samples. Additionally, the extent of cryopreservation-induced damage to spermatozoa can vary depending on the method used. Consequently, differences in the proteomic profiles of the BX and BY samples may also arise from variations in the cryopreservation process.

Therefore, comparing the proteomic profiles of X- and Y-sperm from both dairy and beef semen separately, treated under identical conditions and preferably fresh, would help eliminate potential factors influencing the results, such as the freezing method, sexing technique, and diluent used. Nevertheless, the approach implemented in the present study enabled a more comprehensive characterization of the sperm proteome across distinct breeds, making it valuable for studying proteomic profiles directed towards X selection (for dairy) or Y selection (for beef). Moreover, it contributed to the overall characterization of the sperm (membrane) proteome from *Bos taurus* bulls. Additionally, semen pools were also utilized alongside technical replicates to mitigate the effects of biological individual variation and enhance the validity of our observations.

With this shotgun proteomic analysis, it was possible to detect a total of 130 putative surface proteins of bovine spermatozoa—108 identified in the BU samples, 94 in the BX samples, of which 64 were not identified in the BY samples, and 30 in the BY samples. Contrary to other studies, none of the proteins were exclusively identified in the BY samples (reviewed in [48]). Yet, in some works, it was already possible to observe the identification of a higher number of X-specific proteins compared to Y-specific proteins, as well as higher protein concentrations in unsexed semen compared to sexed semen, as well as in X-semen compared to Y-semen [12,19].

Interestingly, a total of 22 proteins identified in the BX and BY samples were not identified in the BU samples. This discrepancy could be attributed to the sensitivity of the LC-MS/MS, where certain proteins may be masked in the BU samples containing both X-sperm and Y-sperm, possibly due to their low abundance [49]. Furthermore, the unsexed semen was fresh and unaffected by cryopreservation and sperm sexing processes, both known to induce capacitation-like changes in spermatozoa and proteome remodeling [47,50]. Recently, Arunkumar and colleagues identified a total of 156 proteins with differential expression in bull spermatozoa between fresh and cryopreserved states [47]. It has been previously noted that the sperm sexing process also results in varying protein abundances when comparing unsexed and sexed cryopreserved samples [51]. However, among the 22 proteins not identified in the BU samples, none were found in the lists of dysregulated proteins due to cryopreservation or sperm sorting obtained in those studies, except for pyruvate kinase (PKM), since Mostek et al. reported that a protein predicted to be PKM was more abundant in bovine sexed semen [51]. Therefore, future studies should aim to analyze both fresh and cryopreserved samples from multiple breeds to fully capture the variability in protein expression and ensure the robustness of potential biomarkers for sperm sexing.

Moreover, none of the 130 quantified proteins were found in the BC condition where no biotin was added to the spermatozoa, demonstrating that the kit allowed for the elution of a majority of biotinylated proteins. If any non-biotinylated proteins were still eluted, they represented residual amounts, not detected by mass spectrometry.

For a more comprehensive characterization of the 130 quantified proteins, as well as those identified in the BX samples and BY samples, an overrepresentation test was conducted using PANTHER v.18.0 software. The 130 quantified proteins were associated with an overrepresentation of proteins predominantly involved in reproductive and developmental processes, particularly in male reproductive system development and sex differentiation, namely metalloproteases. An enrichment in molecular functions, such as cadherin and calcium ion binding, was also observed. In fact, calcium-dependent adhesion events are involved in the fertilization process in mammals [52]. Proteins related to the sperm principal piece/flagellum and acrosome vesicle, namely involved in sperm motility and organization of the basic structure and assembly of the fibrous sheath, were also overrepresented [53].

In terms of differences between X- and Y-sperm, earlier proteomic studies conducted on bovine unveiled variations in the expression levels of proteins relevant to processes such as energy metabolism, stress response, sperm capacitation, migration velocity, and serine protease activity [13,16,18]. Additionally, differences were observed in proteins related to the cytoskeletal structure of the flagellum (e.g., axoneme, outer dense fibers, and fibrous sheath), as well as in other factors that could contribute to variations in the state, size, and immune reactivity of X- and Y-sperm membranes [15,16]. In the present study, the set of proteins identified in the BX samples exhibited a pattern of overrepresented terms very similar to that observed in the total set of quantified proteins. The BY proteins followed the same trend in terms of cellular component terms, but it was not possible to determine significantly overrepresented biological functions. However, it is noteworthy that in the BY condition, proteins involved in cadherin binding were overrepresented and no enrichment of proteins related to the acrosome vesicle was observed, in contrast to the BX condition.

Among the 130 quantified proteins in our study, 8 have been previously described as upregulated in X-sperm compared to Y-sperm (AKAP3, CALM, FUNDC2, GAPDHS, ODF2, SPACA1, TUBB4B, and DSP) [15,16,17,18,19], and 2 as upregulated in Y-sperm (ATP5F1B and ABCA14) [9,12]. Although in the present study no significant differences were observed in terms of the expression of these proteins between the BX and BY samples, CALM and AKAP3 were identified in one replicate of the BX samples and not in the BY samples, and FUNDC2, GAPDHS, and TUBB4B were identified in two or three replicates of the BX samples and not in the BY samples. According to our bioinformatic analysis, among these five proteins, CALM was the only protein found to be associated with the plasma membrane. However, it is described as an integral component of the plasma membrane and was predicted to be a globular protein, thus not meeting the criteria for further evaluation as a sex-specific cell surface biomarker. SPACA1 and DSP were identified both in the BX and BY samples, and ODF2 was only identified in the BU samples. Conversely to the results of Chen et al. and Laxmivandana et al., in our study, ATP5F1B was detected in two replicates of the BX samples and not in the BY samples, and ABCA14 was identified in three replicates of the BX samples and one of the BY samples [15,18].

Curiously, ADAM2, the only protein found to be significantly upregulated in the BX samples compared to the BY samples, has not been previously identified as upregulated in other studies comparing bovine X-semen and Y-semen. ADAM2 is a protein known to be involved in important processes related to the adhesion and fusion of the spermatozoa and oocyte [54,55,56]. Although it was not identified in the BY samples, caution should be exercised, as this significant difference may be associated with variations in sample quality rather than indicating a sex-specific influence. Further studies are necessary to validate whether this protein is indeed upregulated in X-sperm, using a higher number of samples.

The discrepancies between our study and others in the literature regarding the detection of sex-specific proteins and/or differentially expressed proteins between X- and Y-sperm, such as in the study by Shen et al. (2021), may stem from technical differences in the protein extraction and analysis methods, as well as variations in the samples used [12]. For example, Shen et al. employed a kit designed for plasma membrane protein extraction through centrifugation, whereas the kit used in the present study was aimed at targeting cell surface proteins through biotinylation [12]. Additionally, while our study included sperm samples from different breeds, Shen et al. exclusively used X- and Y-sperm from Holstein bulls [12].

To enhance the likelihood of identifying a potential protein target that is stable and accessible from the cell surface, the mass spectrometry analysis was complemented with a GO analysis and topology prediction for each detected protein. While bioinformatics tools provide valuable insights into membrane-associated protein prediction, they have inherent limitations that can impact accuracy and reliability. These constraints stem from factors such as the complexity of protein topologies, the quality of training datasets, and the challenges of distinguishing similar features. In fact, despite the growing discovery of proteins with non-standard topologies, topology prediction remains the preferred method for large-scale analysis over 3D structure prediction [57]. The software used in the present study for protein topology prediction, DeepTMHMM, stood out in a previous study as the most reliable tool for large-scale topology predictions of alpha-helical and beta-barrel transmembrane proteins with over 80% accuracy, surpassing alternatives like TOPCONS2, CCTOP, DMCTOP, and Phobius [29]. Misclassification of signal peptides as transmembrane segments is also a common issue, but DeepTMHMM resolves this by predicting both topology and signal peptides simultaneously [29]. Moreover, it uses the CD-HIT8 algorithm with a 30% sequence identity cut-off for homology reduction to avoid overfitting and overly optimistic performance estimates [29]. Although most software does not address the prediction of surface-bound proteins, DeepTMHMM also predicts globular proteins and signal peptides, while predicting which parts of the proteins’ sequences are located inside or outside the cell. To verify the surface localization of a protein, flow cytometry or immunocytochemistry assays can be performed using antibodies directed specifically against the extracellular regions of the protein, without permeabilizing the spermatozoa.

After analyzing the possible cellular localization and topology predictions of the 64 proteins detected in the BX samples and not in the BY samples, it was observed that 26 proteins were already associated with the plasma membrane, of which 19 proteins were identified as possibly accessible from the cell surface. Among these, five proteins were considered promising candidates for the development of bovine sperm sexing technologies, as X-sperm targets, namely ADAM2, ATP11C, DG1, MCT1, and PMCA4. They were identified in at least two replicates of the BX samples and not in the BY samples and were predicted to have at least one transmembrane domain. Importantly, these five potential targets were also identified during optimization assays in two additional independent runs using fresh unsexed spermatozoa from other Limousin bulls with similar reproductive history, all provided by Lusogenes (Supplementary Spreadsheet S1.9). The first run involved 24 and 72 million spermatozoa of a single bull, while the second utilized 1000 million spermatozoa of another bull. The protein extraction and shotgun analysis were performed using the same kit and protocols. In these independent assays, all five selected potential targets of bovine X-sperm were identified by at least four peptides when using 72 million spermatozoa. Moreover, when using 24 million spermatozoa, only ATP11C was not identified (other targets identified by at least two peptides), and, when using 1000 million spermatozoa, only DG1 was not identified (other targets identified by at least five peptides) (see Supplementary Spreadsheet S1.9).

**ADAM2** is a sperm surface protein believed to play a role in the adhesion of spermatozoa to the oocyte surface and subsequent fusion [54,55,56]. Furthermore, in *ADAM2* gene knockout mice, spermatozoa retained motility but exhibited functional defects, failing to reach the oviduct, suggesting a critical role of ADAM2 in the migration of spermatozoa to the oocyte [58,59,60]. Additionally, higher ADAM2 levels may correlate with better sperm quality. This observation is supported by a study by Heidari et al., which found that spermatozoa from asthenoteratozoospermic individuals had reduced levels of ADAM2 compared to normozoospermic individuals [61]. ADAM2 localization in the acrosomal region of human spermatozoa [61] and the acrosomal cap of both epididymal and ejaculated camel spermatozoa has been previously described [62]. **ATP11C** is a phospholipid-transporting ATPase localized in the cell membrane, encoded by the X chromosome [63]. It is part of the P4-ATPase flippase complex, which catalyzes the hydrolysis of ATP coupled to the transport of aminophospholipids, phosphatidylserines, and phosphatidylethanolamines from the outer to the inner leaflet of the plasma membrane [64,65]. ATP11C is the primary P4-ATPase found in liver, but its presence was also noted in other tissues, such as testes [63]. While ATP11C deficiency in mice does not impact fertility, it has been described to lead to various abnormalities, including hyperbilirubinemia, hepatocellular carcinoma, anemia, X-linked cholestasis, and loss in B cell development [66,67,68]. **DG1** is also a cell membrane protein and a component of desmosome junctions. It plays a role in the interaction of plaque proteins and intermediate filaments, mediating cell–cell adhesion [69]. It was reported to be reduced in lung cancer patients [70]. Nevertheless, Selvam et al. found this protein uniquely expressed in the spermatozoa of testicular cancer patients compared to fertile healthy men, although at very low abundance [71]. Additionally, Rajamanickam et al. reported that DG1 interacts with the testis-specific isoform of Na/K ATPase in the plasma membrane of bovine sperm during capacitation [72]. **MCT1** catalyzes the rapid transport of various monocarboxylates, including lactate, pyruvate, and ketone bodies, across the plasma membrane [73,74]. MCT1 was already found on the surface of spermatogonia, spermatocytes and spermatids, sperm head, tail, and midpiece, varying between species (reviewed by [74]), as well as in Sertoli and Leydig cells [75,76]. Bernardino et al. demonstrated that the knockout of MCT1 in mice led to alterations in testicular morphology, resulting in impaired spermatogenesis and the complete absence of spermatozoa [77]. Finally, **PMCA4** is a calcium/calmodulin-regulated and magnesium-dependent enzyme responsible for catalyzing the hydrolysis of ATP coupled with the transport of calcium out of the cell. This multi-pass membrane protein is localized in the cell membrane and is believed to play a role in sperm motility by regulating calcium homeostasis [78,79]. In a study conducted with mice spermatozoa, this protein was primarily observed in the sperm tail, with faint staining in the head and midpiece [80]. Another study involving human spermatozoa revealed PMCA4 localization over the acrosome, midpiece, and proximal principal piece [81]. Posh et al. localized PMCA4 mainly in the midpiece of both caput and caudal bovine spermatozoa [82]. They also verified that capacitation did not alter PMCA4 localization [82]. Studies by Schuh et al. revealed that PMCA4 deficiency in mice leads to male infertility due to defective sperm motility, although this loss does not impair in vitro fertilization capacity [78]. This corroborates another study that states that PMCA4 is required for hyperactivated motility but not for non-hyperactivated motility, as the latter is not calcium-dependent [80]. Andrews et al. also hypothesized that PMCA4 interacts with nitric oxide synthases in sperm, preventing nitric oxide production, which is known to induce asthenozoospermia via oxidative stress [81].

The selection of a protein target sharing orthology, conserved sequence, and similar structure across species increases the likelihood that the same antibody can be used for sperm sexing in a wider range of animals, providing the basis for the development of a universal sperm sexing kit for livestock breeding. To assess the potential applicability of the five targets in rabbits, humans, and other commercially important species such as pigs, sheep, and horses, orthology was evaluated between species primarily using the UniProt and Ensembl databases. Orthologs of all targets were identified in the analyzed species, although the orthology inference was considered of low confidence for ADAM2 in rabbits and horses, and for ATP11C in rabbits. Nonetheless, several externalized amino acid sequences of ADAM2 and ATP11C demonstrate high levels of similarity with equivalent regions of the orthologous proteins in cattle. Further research into sequence and structure conservation will help confirm their potential applicability across different species and provide insights for the development of specific and efficient antibodies for sperm sexing applications. Slight discrepancies in orthology identification for the same analyses may arise from the utilization of different information by the NCBI and Ensembl databases to infer orthology. NCBI identifies orthologs by considering both protein sequence similarity and local synteny information, which involves conserving the order of neighboring genes [83]. Ensembl infers orthology from gene trees constructed using one representative protein for each gene in every species within its database [84,85]. Additionally, Ensembl designates the orthology between two mammalian genes as high confidence if the alignment between them achieves a minimum identity percentage of 50%, and either the gene-order conservation score or the whole-genome alignment coverage scores meet the specified threshold of 75 [84].

It is important to note that the exclusion of proteins not associated with the plasma membrane or cell surface, according to our bioinformatic analysis, does not rule out their potential use in sperm sexing. The approach used does not indicate the absence of those proteins in the desired cellular localization; rather, it reflects that they have not yet been described as such in the consulted databases. Notably, the use of a kit targeting cell surface proteins implies that the actual number of potential targets may exceed those established in this study. Moreover, other proteins specific to X- or Y-sperm may still be of interest for sperm sexing, even if not directly linked to the cell surface. This depends, for example, on their functions, such as those related to sperm motility or ATP production. By interfering with their function, especially through inhibition, there is a chance for the selective impact on sperm motility in one of the spermatozoa types, depending on the activation or absence of compensation mechanisms [14,86].

## 5. Conclusions

In this study, a comprehensive shotgun proteomic analysis was conducted on bovine unsexed and sexed spermatozoa, with a specific focus on cell surface proteins. The analysis detected 130 putative membrane proteins of bovine spermatozoa, with 64 proteins exclusive to X-semen. Among these, one protein pivotal for sperm–egg interaction was significantly upregulated in the BX samples compared to the BY samples. Remarkably, over 40% of the quantified proteins were already associated with the plasma membrane and 30% were predicted to be accessible from the cell surface. Among the 19 promising candidates detected in the BX samples and not in the BY samples, ADAM2, ATP11C, DG1, MCT1, and PMCA4 stood out, sharing orthology and certain sets of amino acid sequences of high similarity with humans, rabbits, pigs, sheep, and horses. However, it is important to emphasize that these results are preliminary and require further validation. Independent validation experiments using additional biological replicates, namely of different bovine populations, as well as cross-species comparisons, are essential to confirm the robustness and specificity of these proteins as markers for X-sperm, and to assess the generalizability of these potential markers. Such validation efforts will be crucial for determining whether these proteins can be reliably used as sex-specific sperm surface targets, not only in bovines but across diverse species.

## Figures and Tables

**Table 1:** List of the 19 detected proteins possibly accessible from the cell surface that were identified in at least one replica of BX samples but not in BY samples. No. BX Replicates, the number of BX replicates in which the protein was identified.

Protein ID	Chromosome	Protein Name	Gene Name	Topology	No. BX Replicates
A0A3Q1LK79	17	Uncharacterized protein	*Adam1b*	SP	2
**O77780**	Unplaced	Disintegrin and metalloproteinase domain-containing protein 2	*ADAM2*	SP+TM	3
P09487	2	Alkaline phosphatase, tissue-nonspecific isozyme	*ALPL*	SP	1
**F1N3G6**	X	Phospholipid-transporting ATPase	*ATP11C*	TM	2
Q3T0C6	1	Sodium/potassium-transporting ATPase subunit beta-3	*ATP1B3*	TM	1
**D3K0R6**	16	Plasma membrane calcium-transporting ATPase 4	*ATP2B4*	TM	2
P00829	Unplaced	ATP synthase subunit beta	*ATP5F1B*	GLOB	2
**Q03763**	Unplaced	Desmoglein-1	*DSG1*	SP+TM	2
Q32LB5	5	GLIPR1-like protein 1	*GLIPR1L1*	SP	3
Q27975	23	Heat shock 70 kDa protein 1A	*HSPA1A*	GLOB	2
P0CB32	23	Heat shock 70 kDa protein 1-like	*HSPA1L*	GLOB	2
P34933	Unplaced	Heat shock-related 70 kDa protein 2	*HSPA2*	GLOB	2
Q0VCX2	11	Endoplasmic reticulum chaperone BiP	*HSPA5*	SP	2
P19120	15	Heat shock cognate 71 kDa protein	*HSPA8*	GLOB	2
P31081	22	60 kDa heat shock protein	*HSPD1*	GLOB	3
**Q3MHW6**	3	Monocarboxylate transporter 1	*SLC16A1*	TM	3
Q32PB3	18	Sperm acrosome membrane-associated protein 4	*SPACA4*	SP	2
F1MTV1	4	Hyaluronidase	*SPAM1*	SP	2
A0A3Q1MQE3	Unplaced	Uncharacterized protein	-	SP+TM	1

**Table 2:** The five most promising bovine X-semen targets possibly accessible from the cell surface identified in at least two replicas of BX samples and not in BY samples. Each target is associated with its respective Reference Sequence (RefSeq) identifier, the percentage of protein sequence similarity with that of orthologs from other species, and the encoding chromosome (Chr) for the respective proteins and species. Orthology was determined based on the NCBI database, local synteny, and/or Ensembl BioMart tool information.

Proteins	Bovine		Human			Rabbit			Pig			Sheep			Horse		
	RefSeq	Chr	Similarity (%)	RefSeq	Chr	Similarity (%)	RefSeq	Chr	Similarity (%)	RefSeq	Chr	Similarity (%)	RefSeq	Chr	Similarity (%)	RefSeq	Chr
ADAM2	XP_024841956	27	60.5	XP_054216215	8	59.3	XP_051684794	2	66.1	NP_999122	15	81.3	XP_027818444	26	68.0	XP_023486557	27
ATP11C	NP_001280029	X	92.5	XP_054182860	X	91.6	XP_051687539	U	92.1	XP_020935853	X	96.9	XP_060264232	X	90.1	XP_023489520	X
PMCA4	XP_024831810	16	89.8	NP_001675	1	89.6	XP_002717626	16	96.3	XP_020918854	9	97.6	XP_060252228	12	93.7	XP_001488333	5
DG1	NP_776470	24	81.0	NP_001933	18	80.6	XP_002713529	9	85.0	NP_001030612	6	94.9	XP_004020502	23	82.6	XP_005612875	8
MCT1	XP_059738538	3	85.2	XP_054194429	1	87.6	XP_051712113	13	91.2	XP_020944110	4	97.4	XP_060268867	1	89.0	XP_023495965	5

## Data Availability

The original contributions presented in the study are included in the article/Supplementary Materials, further inquiries can be directed to the corresponding author.

## Supplementary Materials

The following supporting information can be downloaded at: https://www.mdpi.com/article/10.3390/ani15040484/s1, Spreadsheet S1: mass spectrometry data (S1.1–S1.4), results of the bioinformatic analysis (S1.5–S1.8), and the LC-MS/MS data regarding the identification of the five targets in previous independent runs of fresh Limousin unsexed semen (S1.9); File S1: alignment of the residues of ADAM2 and ATP11C predicted to be outside the cell in both rabbits and bovines.
