# Supplementary material for: The Surface Proteome of Bovine Unsexed and Sexed Spermatozoa
Source: Animals (Basel). 2025 Feb 8;15(4):484. doi: 10.3390/ani15040484 (PMC11852025; doi:10.3390/ani15040484)
Supplement: Supplementary file 1 [file animals-15-00484-s001.zip › File S1. ADAM2 & ATP11C_Outside sequence similarity.pdf]

ADAM2

Bovine (O77780) and Rabbit (XP_051684794) - 62.78%

```
Bov_Out1 D D N S E R L R V Q F T V P E K I R S T S S G G V E T H V S Y I I L I E G K T Y T V N L M Q K A F L P H N F R V Y G Y S G T G S 64
Rab_Out1 - - - - - - - - - - - - - - - - - - - - - - - - - - - - - - - - - - - - - - - - - - - - M Q K T F L P R D F R V Y G Y D S T G I 20

Bov_Out1 M K P L E H E F Q N F C Y Y Q G Y I E G Y P N S M A I I S T C T G L R G L L Q F E N V S Y G I E P L E P S I G F E H M V Y Q I K 128
Rab_Out1 M K D L E Q Q F Q N F C Y Y Q G F V E G Y P N S M V I V S T C T G L R G L L Q F E N V T Y G I E P L E S S I G F E H V I Y Q V K 84

Bov_Out1 P R D S S S S V Y T E R E I E L R E K P Y K I Q N V E P L P D F S Q Y I E M H I V V E K D L Y N H M G A D T T V V I Q K I F Q L 192
Rab_Out1 N K N E S I S L Y A E K E V E F R D L P Y K V Q S V Q P - R E F S Q Y I E M H V V V E K N L Y K H M G S D T A V V S Q K I F Q L 147

Bov_Out1 T G L T N A I F T S L N I T V I L S S L E L W I D E N K I P V T G D A N E L L H R F V K W K R S Y L V L R P H D M A F L L V Y R 256
Rab_Out1 I G L T N A V F T S F N I T I I L S S L E L W I D E N K I S T T G D A N E L L Y R F L K W K K S Y L V L R P H D V A F L L V Y R 211

Bov_Out1 E K S N Y I G A T F Q G R M C D K H Y G G G V A L H S S T I S L E S L A V I I A Q L L S L S M G I P Y D D I N K C H C P G D V C 320
Rab_Out1 E I A K Y V G A T F Q G K M C D I N Y S G G V A L H P K T I S L E S L A I I L A Q L L S L S M G I A Y D D I N K C K C P G S V C 275

Bov_Out1 I M N P A A V H S S G V K L F S N C S V E D F L R F I S K P K S Q C L Q N Q P R L D P T Y K S - A V C G N G K V E E G E Q C D C 383
Rab_Out1 I M N P E A I H S S G V K I F S N C S I E D F S R F I S K Q K S Q C L Q N L P R L E P F Y K Q D A V C G N S I V E A G E M C D C 339

Bov_Out1 G N K K A C D A L P D T C C V A D T C R F Q P G S A C D T G L C C E S C A F I P K G H I C R G S T D E C D L H E Y C N G S S A A 447
Rab_Out1 G T A E E C G R A V P I C C N S A T C R L E V G S Q C A E G E C C E N C T F K E R G Q S C R P P V G E C D L F E Y C N G T S A L 403

Bov_Out1 C Q E D V Y V Q D G H P C G Q N Q W L C I S G I C V D G I K Q C F D I F G E G T S Y A P A E C F Q R L N S M N D L S G N C G V T 511
Rab_Out1 C P D D I V I Q N G H P C G E N Q W I C V D G S C I S P P E Q C K S I F G E E V E G G T P E C Y E E L N A M A D I S G N C G I T 467

Bov_Out1 P T G F T P C T S E N V R C G K L L C T Y D K R E V I S V E N A S V M Y S N I N G K I C I G L H Y E Y G N E D E G M M W V K D G 575
Rab_Out1 H D G Y K K C E S G N R K C G K L M C K H T T E N I I D I K N A T I I Y A N V S G S I C L S L E Y K P D H L D A A K M W V P N G 531

Bov_Out1 T V C G E S K I C Q N K Q C V D S S F L N Y D C N P E K C N N Q G V C N N K K H C H C N P S Y L P P N C E H S A P G W E G G S I 639
Rab_Out1 A V C G S N K I C R D K A C V E T T Y V N L G C T L Q N C N N Q G I C N S L Q H C H C N P T F L P P N C S A V D E R W A G G S V 595

Bov_Out1 D S G N F P P S E P P T G G P A F T D V G T T P L A - - - - - E S R Y I E N V Y R S - K P T R W P 682
Rab_Out1 D S G N F Q G G G V P G I G - G L T S V G T P P F I P G V P G V R R Y M E D V Y Q T A K P T R - - 641
```

ATP11C

Bovine (F1N3G6) and Rabbit (XP_051687539)

```
Bov_Out1 D T P T S P I T S 88.89%
Rab_Out1 D T P T S P V T S

Bov_Out2 K Y V W Q S T P H N D E P W Y N Q K T Q K E R E T W K V L K M F T D 97.06%
Rab_Out2 K Y V W Q S T P H N D E P W Y N Q K T Q K E R E T L K V L K M F T D

Bov_Out3 C G F S Q Q P L Y D A 100%
Rab_Out3 C G F S Q Q P L Y D A

Bov_Out4 Q T T S L D E N G K V Y G N 85.71%
Rab_Out4 Q T A S L E E N G K V Y G N

Bov_Out5 I W P F L K Q Q R M Y F V F A Q 100%
Rab_Out5 I W P F L K Q Q R M Y F V F - -
```
